# Preparation of Asymmetric Al_2_O_3_-SiO_2_ Janus Nanoparticles in Aqueous Phase and Its Interfacial Property

**DOI:** 10.3390/ma17061251

**Published:** 2024-03-08

**Authors:** Xinli Jia, Peiwen Xiao, Liqiang Yang, Jianhui Luo, Meiying He, Pingmei Wang, Bo Jiang, Bo Xiao

**Affiliations:** 1 Key Laboratory of Green Chemistry & Technology, Ministry of Education, College of Chemistry, Sichuan University, Chengdu 610064, China; jiaxl@stu.scu.edu.cn (X.J.); jiangbo@scu.edu.cn (B.J.); 2 Research Institute of Petroleum Exploration & Development (RIPED), Petro China, Beijing 100083, China; xiaopw@petrochina.com.cn (P.X.); luojh@petrochina.com.cn (J.L.); wangpm@petrochina.com.cn (P.W.); 3 Key Laboratory of Nano Chemistry (KLNC), China National Petroleum Corporation (CNPC), Beijing 100083, China; 4 PetroChina, Beijing 100007, China; yangliqiang@petrochina.com.cn; 5 China Chengda Engineering Co., Ltd., Chengdu 610041, China; hemeiyingscu@outlook.com

**Keywords:** Janus nanoparticles, dumbbell-like, surface modification, surfactants, aqueous phase

## Abstract

In this study, asymmetric Al_2_O_3_-SiO_2_ Janus nanoparticles with a dumbbell-like structure were synthesized by a facile chemical process in the aqueous phase. Prior to synthesis, Al_2_O_3_ nanoparticles in hydrosol were amino-modified using 3-aminopropyl triethoxysilane (KH550) and then carboxyl acid-functionalized using a ring-opening reaction of the amine functions with succinic anhydride, imparting unique anionic properties to the Al_2_O_3_ end. SiO_2_ nanoparticles were rendered hydrophobic through modification with hexamethyldisilazane (HMDS) and further functionalized with 3-chloropropyl triethoxysilane (KH230). The two nanoparticle hydrosols were then mixed, and the asymmetric Al_2_O_3_-SiO_2_ Janus nanoparticles were synthesized via the reaction between the –NH_2_ and −CH_2_Cl groups. The prepared Janus nanoparticles were primarily characterized by dynamic light scattering (DLS), Zeta potential (ZP), and transmission electron microscopy (TEM). The results indicated that about 90% of the modified Al_2_O_3_ and SiO_2_ nanoparticles were covalently coupled in a one-to-one manner to form the dominant dumbbell-like structure. These Janus nanoparticles exhibit amphiphilic properties, making them highly promising surfactants for emulsifying oil–water mixtures.

## 1. Introduction

Alumina (Al_2_O_3_) and silica (SiO_2_) are two prevalent types of inorganic nanomaterials with high surface area, porosity, and surface functionalities that can be tailored for specific applications. Their abundant hydroxyl groups on the surface provide ample opportunities for surface chemical modifications. By undergoing surface modification reactions, various chemical groups such as carboxylic acids or amino groups can be introduced onto the surfaces of these nanoparticles. In recent literature, various methods such as the sol–gel method, hydrothermal method, co-precipitation method, and mechanical mixing method have been employed to combine Al_2_O_3_ and SiO_2_ into composite materials to overcome the limitations of their individual use. This amalgamation aims to enhance several key properties including mechanical strength, thermal stability, chemical resistance, electrical properties, and surface properties [1,2,3,4], but the Al/Si components in these Al_2_O_3_-SiO_2_ composites are not separated. Therefore, an important research question involves developing a method to obtain Al_2_O_3_-SiO_2_ nanoparticles with separated Al and Si components. In particular, the Al_2_O_3_ and SiO_2_ parts, except for the connecting region, are relatively independent, and the synthesis or modification of the two parts is also carried out separately, resulting in weak chemical interactions between two chemically distinct regions. Until now, few studies have been reported in the literature on this idea, which inevitably draws our attention to the fascinating structure of “Janus nanoparticles”.

Janus nanoparticles were first proposed as anisotropic nanomaterials by Pierre-Gilles de Gennes in 1991 [5,6,7]. Since then, research on Janus nanoparticles has been thriving, and they have received considerable attention due to their unique physical, chemical, and biological properties [8]. Different techniques such as masking, bottom-up assemblies, and controlled phase separation have been employed to synthesize Janus nanoparticles [9,10], and various Janus architectures have been created, such as cylindrical-shaped [11], dumbbell-shaped with asymmetric or snowman characters [12,13,14,15,16,17], mushroom-shaped [18,19], bowl-like [20,21], crescent-shaped [22,23], and half-raspberry-shaped structures [24]. Among these, dumbbell-like nanoparticles, as a prominent subset of Janus nanoparticles, have attracted significant interest due to their unique structure and excellent performance in various application domains, including drug delivery, catalysis, sensing, and advanced materials.

Existing research on dumbbell-like Janus nanoparticles has been classified into three types based on the materials of the two lobes, namely organic polymer double spheres, polymer–inorganic double spheres, and inorganic material double spheres. For example, Abdollahi et al. studied the fast and facile light-induced morphology transition of functional latex nanoparticles from spherical polymer nanoparticles to dumbbell-like particles by surface-incorporation of coumarin derivatives via post-polymerization modification methods [25]. Reculusa et al. synthesized colloidal particles with a dumbbell-like structure combining both organic and inorganic parts through a seeded emulsion polymerization process [26]. Guo et al. synthesized one-to-one dumbbell-type heterodimer Au-Fe_3_O_4_ nanoparticles by etching Fe_3_O_4_ NPs with HAuCl_4_ [27]. Dumbbell-like Janus Au-SiO_2_ nanoparticles synthesized through a Linker-Free approach in the ethanol phase were described by Hu et al. [28]. However, all these reported cases of dumbbell-like Janus nanoparticles were often close to micrometers in size and typically involved various types of organic polymers. These methods involve complex chemical reactions and multi-step processes, leading to increased complexity in the synthesis procedure. In addition, the use of costly reagents makes large-scale production economically unfeasible. In particular, certain synthesis methods may use hazardous chemicals or generate toxic byproducts, posing environmental risks and safety concerns. Thus, it is challenging and interesting to develop convenient, green and environmentally friendly methods for preparing Janus nanoparticles with dumbbell structures using inorganic nanomaterials rather than organic polymers. Recently, we reported a facile method for preparing amphiphilic dumbbell-like SiO_2_ nanoparticles by using two different silane coupling agents [29,30]. Even though the transformation from single nanospheres to dumbbell-like nanospheres is incomplete, this method often requires fewer expensive reagents and equipment, resulting in lower production costs compared to other methods. Hence, it can be applied to produce dumbbell-like Al_2_O_3_-SiO_2_ Janus nanoparticles; so far, few investigations have studied the preparation of these inorganic Janus nanoparticles.

In this work, asymmetric Al_2_O_3_-SiO_2_ Janus nanoparticles with dumbbell-like structure were synthesized through a simple method in an aqueous phase, where hydrophilic Al_2_O_3_ nanoparticles containing −NH_2_ groups reacted with hydrophobic SiO_2_ nanoparticles containing −CH_2_Cl groups. In this process, two nanoparticles in Janus nanoparticles were separately modified in advance and Al_2_O_3_-SiO_2_ Janus nanoparticles with well-defined anisotropic properties can be obtained after coupling. Notably, this coupling reaction resulted in the formation of Janus nanoparticles with anionic properties at Al_2_O_3_ lobe compared to previous studies. The obtained nanoparticles, containing asymmetric components at both ends, were analyzed using various characterization techniques. This template-free method operates in a mild aqueous phase, rendering it suitable for large-scale production of dumbbell-like nanoparticles based on homogeneous inorganic materials. These nanoparticles exhibit a distinct dumbbell-like structure with varying hydrophilicity at each end, conferring upon them “amphiphilic Janus” properties. As such, this kind of Janus nanoparticle is anticipated to serve as a promising candidate material for emulsifying oil–water systems.

## 2. Materials and Methods

### 2.1. Chemicals

Nano SiO_2_ hydrosol (pH = 5, 30 wt%) and nano Al_2_O_3_ hydrosol (pH = 5, 20 wt%) were purchased from Xuancheng Jingrui New Materials Co., Ltd. (Xuancheng, China). 3-aminopropyl triethoxylsilane (KH550, 98%), 3-chloropropyl triethoxylsilane (KH230, 98%), and succinic anhydride (SA, 99%) were purchased from Chengdu Kelong Regents Company (Chengdu, China). All the above chemicals were used as received without any further purification. The deionized water was produced in our laboratory by using an ultrapure water machine with a resistance of 18.25 MΩ.cm (UPH-I-10T, Ulupure Ultrapure Technology Co., Ltd., Chengdu, China).

### 2.2. Procedures

#### 2.2.1. Preparation of Carboxylic Acid-Functionalized Al_2_O_3_ Nanoparticles

A glass beaker was charged with 40 g nano Al_2_O_3_ hydrosol, which was then diluted with deionized water to a concentration of 5 wt%. To functionalize the Al_2_O_3_ nanoparticles with KH550, different amounts of KH550 were added in the above system with vigorous stirring for 4 h using a water bath at 40 °C, with the KH550/Al_2_O_3_ mass ratio ranging from 10 to 25 wt%. The resulting modified Al_2_O_3_ nanoparticles were labeled as A_10KH550_, A_15KH550_, A_20KH550_, and A_25KH550_, respectively. Next, SA was added to each modified hydrosol at n(KH550): n(SA) = 2:1. The mixture was stirred overnight, and the resulting carboxylic acid-functionalized Al_2_O_3_ nanoparticles were labeled as A_10KH550_-COOH, A_15KH550_-COOH, A_20KH550_-COOH, and A_25KH550_-COOH, respectively.

#### 2.2.2. Preparation of HMDS-Modified SiO_2_ Nanoparticles

First, 40 g SiO_2_ hydrosol was added to a glass beaker, diluted with deionized water to a concentration of 5 wt%, and heated up to 40 °C using a water bath. The SiO_2_ nanoparticles were then modified by adding HMDS into the system under continuous stirring at 40 °C for 4 h, where the mass ratio of HMDS/SiO_2_ ranged from 10 to 25%. Finally, the modified SiO_2_ hydrosols were aged for 3 days at room temperature (25 ± 2 °C). The SiO_2_ nanoparticles after modification were recorded as S_10HMDS_, S_15HMDS_, S_20HMDS_, and S_25HMDS_, respectively.

#### 2.2.3. Preparation of Asymmetric Al_2_O_3_-SiO_2_ Janus Nanoparticles

Before coupling the two types of nanoparticles, a small quantity of KH230 (20 μL for 1 g SiO_2_) was added to the HMDS-modified SiO_2_ nanoparticles and stirred for 6 h in a water bath at 40 °C. Then, carboxylic acid-functionalized Al_2_O_3_ nanoparticle hydrosols with a predetermined mass ratio were added into the reactor, and the mixture was then placed in a water bath at 40 °C with continuous stirring for 4 h. The resulting product was aged for 3d at room temperature (25 ± 2 °C). For simplicity, the prepared asymmetric Al_2_O_3_-SiO_2_ Janus nanoparticles obtained by coupling A_25KH550-_COOH and S_25HMDS-KH230_ are denoted as A_25KH550_-COOH @ S_25HMDS-KH230_.

#### 2.2.4. Emulsification Experiment

The various emulsions were prepared according to the literature [29]: 0.05 g asymmetric Al_2_O_3_-SiO_2_ Janus nanoparticles were added to a 10 mL glass sample vial. Next, 5 mL oil and 4 mL water were added, and the resulting mixture was emulsified in a high-speed homogenizer (IKA T18 digital ULTRA-TURRAX, Shanghai Ke Huai Instrument Co., Ltd., Shanghai, China) at 3000 rpm for 10 min at room temperature (25 ± 2 °C). Four systems were investigated, namely cyclohexane–water, toluene–water, silicone oil–water, and vegetable oil–water. The emulsion stability with time was assessed by monitoring the variation in the emulsion complete phase separation.

### 2.3. Characterization of Samples

Particle size and distribution of the samples were analyzed by dynamic light scattering instrument (DLS, Malvern Zetasizer Nano ZS90, Malvern, UK, and wavelength of 633 nm) at 25 °C. The zeta potential measurements of samples were also performed with Malvern Zetasizer Nano ZS90. The chemical composition of samples was analyzed by Fourier transform infrared spectroscopy (FTIR, Bruker Tensor 27, Ettlingen, Germany) using the KBr pellet technique in transmission mode. The morphology of samples was obtained by a transmission electron microscope (TEM, FEI Tecnai G2 F20, Hillsboro, OR, USA) operated at 200 kV. EDX characterizations were performed using JEM-F200 (JEOL, Tokyo, Japan) to analyze the elemental composition of the samples. The samples were prepared on a holey carbon-coated copper grid by placing a drop of the sample. Water contact angles (WCAs) of samples were recorded by a contact angle meter (ChengHui Instruments, Jiangsu, China, JGW-360B) using deionized water as the probe liquid and the droplet usage for each test was 3.0 μL. The thermogravimetry measurements were made with a thermoanalyzer instrument (TGA, DTG-60H, Tokyo, Japan) using standard alumina crucibles. The samples were heated at a rate of 10 °C/min from room temperature to 600 °C in a nitrogen atmosphere. X-ray photoelectron spectroscopy (XPS) was carried out using an XPS instrument (XSAM 800, Manchester, UK) equipped with a monochromatized Al Kα X-ray source to analyze various samples. The C1s peak at 284.6 eV was utilized for the calibration of the binding energy values. The emulsion stability was further studied by the Turbiscan Lab^Expert^ (Formulaction, France) using the multiple light scattering technique and the various samples of emulsion acquired at intervals of 5 min over 3 h at 25 °C.

## 3. Results and Discussion

### 3.1. Synthetic Route of the Asymmetric Al_2_O_3_-SiO_2_ Janus Nanoparticles

**Figure 1:**
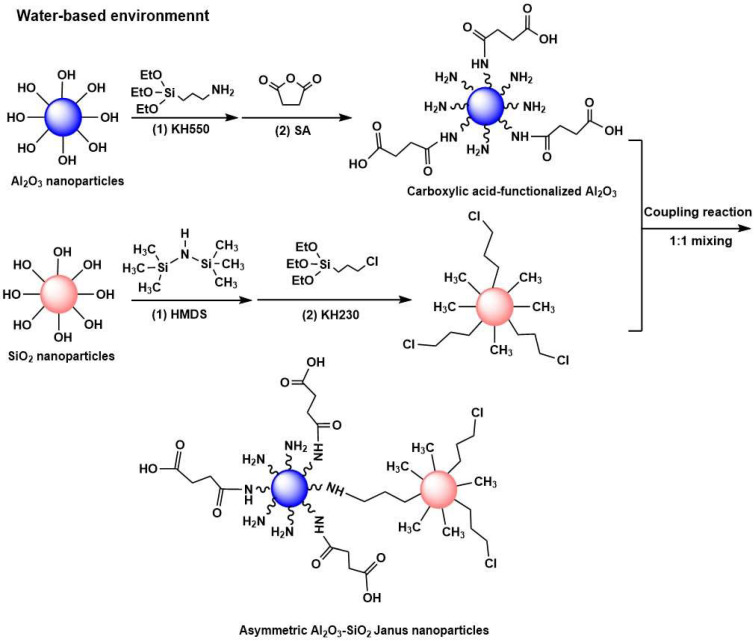
Schematic illustration of the preparation process of asymmetric Al_2_O_3_-SiO_2_ Janus nanoparticles.

The surface of Al_2_O_3_ and SiO_2_ can be easily modified with silane coupling agents due to the presence of hydroxyl groups on the surface to further bridge the organic/inorganic components. Figure 1 shows the synthetic routes used to obtain asymmetric Al_2_O_3_-SiO_2_ Janus nanoparticles, consisting of three steps. Firstly, amino-functionalized Al_2_O_3_ nanoparticles were prepared by a silanization process with KH550 and carboxylic acid-functionalized Al_2_O_3_ nanoparticles were then synthesized by a ring-opening linker elongation reaction of the −NH_2_ functions with SA. In the second step, the surface of SiO_2_ nanoparticles was grafted with −CH_3_ groups through a silanization process with HMDS. Lastly, the hydrophobic modified SiO_2_ nanoparticles with HMDS were further functionalized with KH230, and the two types of nanoparticles with different wettability were then covalently linked together on a one-to-one basis via the substitution reaction between the −NH_2_ and −CH_2_Cl groups on the surface of the nanoparticles. The entire process was carried out in water-based environment.

### 3.2. Carboxylic Acid-Functionalized Al_2_O_3_ Nanoparticles

**Figure 2:**
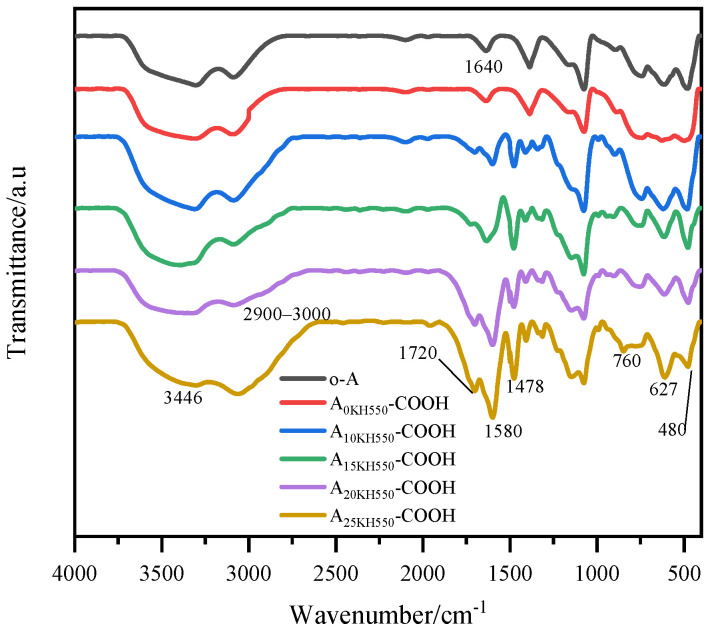
FTIR spectra of carboxylic-functionalized Al_2_O_3_ nanoparticles.

The solid samples for FTIR analysis were obtained by drying the modified Al_2_O_3_ hydrosol at 100 °C and washing it thoroughly with ethanol and water to remove unreacted SA. Figure 2 exhibits the FTIR spectra of carboxylic acid-functionalized Al_2_O_3_ nanoparticles in the range 400–4000 cm^−1^. The peaks at 3446 and 1640 cm^−1^ were attributed to the stretching and bending vibrations of the hydroxyl group, respectively, while the peaks at 760, 627, and 480 cm^−1^ were caused by the vibration of the Al-O bonds of Al_2_O_3_ nanoparticles. After modification, the peak at about 2900–3000 cm^−1^ can be ascribed to the absorption of −CH_2_−, which corresponds to the −CH_2_− groups of KH550 and SA. A new absorption peak of carboxylic acid-functionalized Al_2_O_3_ nanoparticles appeared at 1720 cm^−1^ corresponding to O=C-OH [31,32]. Furthermore, the peak at 1580 cm^−1^ was attributed to the vibration of the amide bond, suggesting that the conjugation of amino-functionalized Al_2_O_3_ nanoparticles with SA was achieved through a ring opening linker elongation reaction of the amine functions with SA. In addition, a control experiment was conducted under the same conditions by mixing the Al_2_O_3_ hydrosol with SA (A_0KH550_-COOH), but without KH550. As shown in Figure 2, the absorption peaks of carboxyl and amide were not observed in the FTIR of A_0KH550_-COOH, indicating that the reaction between SA and Al_2_O_3_ nanoparticles did not occur, and the unreacted SA can be effectively washed off.

The −COOH groups on the surface of Al_2_O_3_ nanoparticles were quantitatively titrated using the simplest acid–base titration method and the detailed titration process is provided in the Supplementary Materials Text S1. The grafting ratio of SA on the surface of modified Al_2_O_3_ nanoparticles was calculated using Equation (1), and the results are presented in Table 1. In the control experiment, the grafting ratio of SA in A_0KH550_-COOH was negligible, suggesting the absence of −NH_2_ groups on the surface of Al_2_O_3_. The grafting ratios of SA in carboxylic acid-functionalized Al_2_O_3_ nanoparticles increased from 17% to 44% with the simultaneous increase in KH550 and SA. The amount of KH550 used was in excess compared to SA in the reaction system to ensure that there were remaining −NH_2_ groups on the surface of Al_2_O_3_ nanoparticles for subsequent coupling reactions. The conversion rate (CR) of SA in different samples was calculated according to Equation (S1), ranging from 76 to 81%, indicating a relatively complete reaction between SA and amino-functionalized Al_2_O_3_ nanoparticles.

(1) $${grafting\;ratio\;}_{SA}^{\;a}=\frac{{n_{SA}\times M}_{SA}}{1000\times m_{sample}}\times 100\%\;$$

where $n_{SA}$ in 1 g of sample is explicitly given by Equation (S2) and the ${grafting\;ratio\;}_{SA}^{\;a}$ represents the grafting ratio of SA calculated by acid–base titration, namely the proportion of the mass of SA involved in the reaction to the mass of sample. $n_{SA}$ is the molar mass of SA, $M_{SA}$ is the molecular weight of SA and $m_{sample}$ is the mass of the sample.

**Figure 3:**
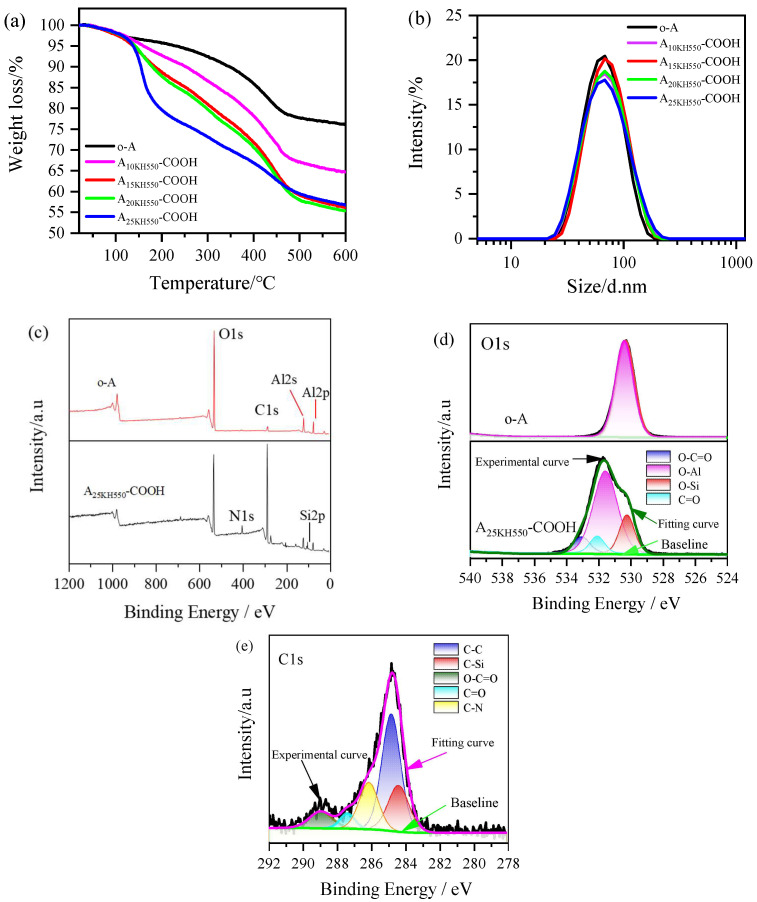
TGA curves (**a**), particle size distribution (**b**), and XPS spectra (**c**–**e**) of Al_2_O_3_ nanoparticles before and after carboxylic acid functionalization.

Figure 3a shows the TGA curves of Al_2_O_3_ nanoparticles before and after modification. The weight loss at temperatures lower than 140 °C is attributed to the evaporation of physis orbed water from the surface of o-A. The weight loss of o-A in the range of 140–600 °C is around 20%, which corresponds to the slow condensation of Al-OH. According to Equation (2) [33], the −OH group content can be calculated as 22.22 mmol per gram of Al_2_O_3_ at T_0_. After modification, the weight loss curves of four samples at temperatures lower than 140 °C are similar. However, these curves in the range of 140–600 °C are always below the curve of O-A, mainly due to the decomposition of organic components grafted on the surface of Al_2_O_3_ nanoparticles and the condensation of residual −OH groups at higher temperatures. As the samples were washed with ethanol and deionized water beforehand, the weight loss of the physical adsorption SA could be neglected. For inorganic oxide nanoparticles, surface modification was achieved through the reaction between the modifier and the −OH groups on the surface of the nanoparticles. Therefore, the grafting ratio of the modifier can be defined as the ratio of the mass of the modifier grafted to the nanoparticle surface to the total mass of the −OH groups of the nanoparticle [34]. According to Equation (3), the total grafting ratios of KH550 and SA on the surface of the four modified Al_2_O_3_ nanoparticles were calculated to be approximately 50%, 58%, 69%, and 77%, respectively.

(2) $$n_{OH}=2n_{H_{2}O}=\frac{2(W_{T_{0}}-W_{T_{final}})}{100M_{H_{2}O}}$$

(3) $${grafting\;ratio\;}_{SA}^{\;b}=\frac{m_{modifier}}{m_{OH}}\times 100\%$$

where $W_{T_{0}}-W_{T_{final}}$ is the weight loss (wt.%) in the temperature region from *T_0_* to $T_{final}$, $M_{H_{2}O}$ is the molecular weight of H_2_O, the ${grafting\;ratio\;}_{SA}^{\;b}$ represents the grafting ratio of SA determined by TGA analysis, $m_{modifier}$ is the mass of modifiers grated on the surface of nanoparticles and $m_{OH}$ is the mass of the −OH group in nanoparticles.

Based on the TGA curve of KH550-modified Al_2_O_3_ nanoparticles shown in Figure S1, the grafting ratios of KH550 in A_10KH550_, A_15KH550_, A_20KH550_, and A_25KH550_, calculated using Equation (3), were determined to be 37%, 40%, 48%, and 53%, respectively. Consequently, the grafting ratios of SA on the surface of Al_2_O_3_ nanoparticles were calculated as 13%, 18%, 21%, and 24%, respectively. Note that the grafting ratio of SA increased with the increase in the amount of modifier using both methods, but the grafting ratio of SA calculated by the thermogravimetric method was found to be lower than that calculated by the titration method. This difference can be attributed to the fact that the −OH groups measured by thermogravimetric analysis include both surface and internal −OH groups. Consequently, the measured content of −OH groups is higher, resulting in a lower grafting ratio.

The particle size distributions of the original Al_2_O_3_ nanoparticles (o-A) and carboxylic-functionalized Al_2_O_3_ nanoparticles were shown in Figure 3b. The average size of o-A was 61 ± 1 nm and the polydispersity index (PDI) value was below 0.13, indicating good dispersity. The average particle sizes of the carboxy-functionalized products (A_10KH550_-COOH, A_15KH550_-COOH, A_20KH550_-COOH, and A_25KH550_-COOH) were 62 ± 1, 64 ± 1, 61 ± 1, and 62 ± 1 nm, respectively. It can be observed that the average particle size of the resulting products did not change significantly compared to o-A. The PDI values for the carboxy-functionalized products ranged from 0.15 to 0.17, which indicates that Al_2_O_3_ nanoparticles before and after surface modification also have good dispersity in hydrosol. Furthermore, the carboxylic acid-functionalized Al_2_O_3_ nanoparticle hydrosols were found to be stable for more than two months.

XPS analysis of Al_2_O_3_ nanoparticles before and after modification was performed to confirm the change in chemical bond on the surface of nanoparticles, as shown in Figure 3c–e. The electron core level XPS spectra of o-A, namely Al2p, Al2s, and O1s, indicate the presence of aluminum and oxygen components in the sample. The weak carbon signal peak in the XPS full survey spectra is attributed to the carbon pollution of the instrument itself. For the carboxylic acid-functionalized Al_2_O_3_ nanoparticles (A_25KH550_-COOH), in addition to Al and O elements, N and Si signal peaks also appeared, and the signal peak of C was significantly enhanced, indicating that KH550 was grafted on the surface of Al_2_O_3_ nanoparticles (Figure 3c). The O1s spectra signal peak in o-A is about 531.1 eV, while the O1s spectra of A_25KH550_-COOH can be fitted into four peaks at 531.8 eV, 530 eV, 532 eV and 533.1 eV, corresponding to O-Al, O-Si, C=O and O-C=O, respectively (Figure 3d). The shift of O-Al binding energy in A_25KH550_-COOH towards the left may stem from the reduction in electron density on the surface after the chemical reaction between the −OH groups on the surface of Al_2_O_3_ and the modifier. The C1s spectra of A_25KH550_-COOH can be deconstructed into five individual peaks, each representing a separate carbon bond, namely C-C (284.8 eV), C-Si (284.2 eV), C-N (286.6 eV), C=O (287.4 eV) and O-C=O (289.0 eV) [35] (Figure 3e), further indicating the successful preparation of carboxylic acid-functionalized Al_2_O_3_ nanoparticles.

### 3.3. Hydrophobic Modification of SiO_2_ Nanoparticles by HMDS

**Figure 4:**
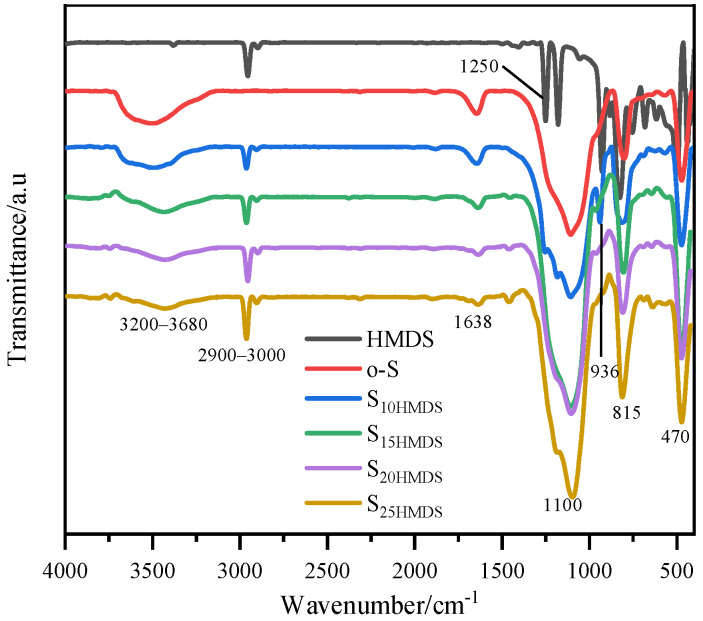
FTIR spectra of HMDS and SiO_2_ nanoparticles before and after modification with HMDS.

The SiO_2_ powders obtained by SiO_2_ hydrosol were washed with ethanol and deionized water using a similar protocol as that of the Al_2_O_3_ nanoparticles prior to their FTIR and TGA analysis. Figure 4 shows the FTIR spectra of SiO_2_ nanoparticles before and after modification with HMDS. For the original SiO_2_ nanoparticles (o-S), the broadband at 3200–3680 cm^−1^ is associated with the asymmetric stretching vibration of O-H bonds in −OH groups bonded on the surface of SiO_2_, while the peak at 1638 cm^−1^ corresponds to the bending vibration of O-H bonds. The characteristic peaks of 1100, 815, and 470 cm^−1^ correspond to asymmetric stretching vibration, symmetric stretching vibration, and bending vibration of Si-O-Si bonds of SiO_2_ nanoparticles. After modification with HMDS, several new peaks appeared in the SiO_2_ nanoparticles. The asymmetric and symmetric stretching vibrations of C-H bonds in −CH_3_ groups united on the surface of SiO_2_ nanoparticles were observed at 2900–3000 cm^−1^. Additionally, the peaks at 1250 and 936 cm^−1^ correspond to the stretching vibration and bending vibration of −CH_3_ groups directly connected with Si. As the amount of HMDS added increases, the characteristic absorption of −OH groups at 3200–3680 cm^−1^ and 1638 cm^−1^ gradually weakened, indicating that HMDS has been grafted onto the silica surface in the form of new Si-O-Si bonds, and the polar −OH groups on silica surface are progressively replaced by nonpolar −CH_3_ groups.

**Figure 5:**
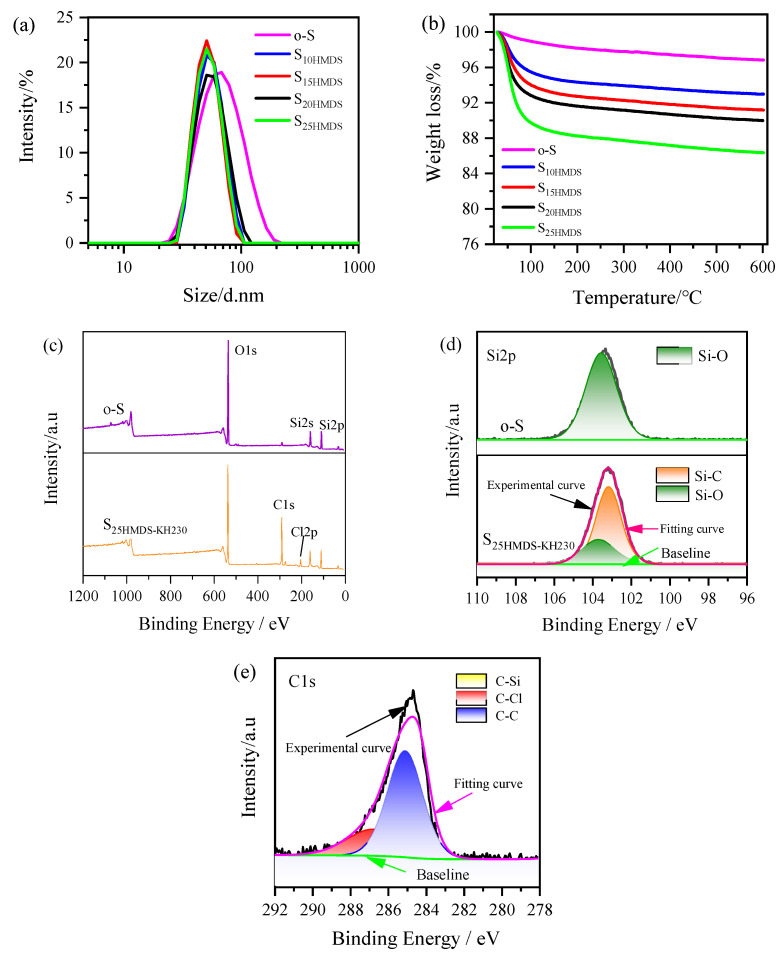
Particle size distribution (**a**), TGA curves (**b**), and XPS spectra (**c**–**e**) of hydrophobic modification of SiO_2_ nanoparticles.

As presented in Figure 5a, the average particle size of o-S is 60 ± 1 nm with a PDI value of 0.19. After modification with HMDS, the average particle sizes of S_10HMDS_, S_15HMDS_, S_20HMDS_, and S_25HMDS_ were measured to be 52 ± 2, 50 ± 1, 53 ± 2, and 51 ± 1 nm, respectively. Note that the modification process caused a change in the particle size and distribution, resulting in a reduction in the average particle size from 60 nm to 50 nm, which suggests that the agglomeration of SiO_2_ nanoparticles was reduced due to the grafting of the −CH_3_ groups onto the surface of SiO_2_ nanoparticles during modification. With an increase in the amount of HMDS added, the PDI of the resulting products decreased, with values of 0.04, 0.01, 0.07, and 0.05, respectively. This indicates that surface-modified SiO_2_ nanoparticles with HMDS exhibited good monodispersity in the aqueous phase, as confirmed by the narrow distribution of modified SiO_2_ nanoparticles shown in Figure 5a compared with o-S. Furthermore, the modified SiO_2_ hydrosol was quite stable during the aging process, lasting for up to three months.

The TGA curve of SiO_2_ nanoparticles before and after modification with HMDS is illustrated in Figure 5b. The weight loss below 100 °C for o-S is mainly due to the physically adsorbed water on the silica surface. Between 100 and 600 °C, o-S showed a weight loss of about 2%, which can be assigned to the condensation of silanols. The −OH group content of the SiO_2_ nanoparticle was calculated as 2.22 mmol using Equation (2) in Section 3.2, which is lower than that of Al_2_O_3_ nanoparticles. The weight loss of S_10HMDS_, S_15HMDS_, S_20HMDS_, and S_25HMDS_ can be ascribed to the thermal decomposition of HMDS chemically bonded to the surface of SiO_2_ and the condensation of residual hydroxyl groups at higher temperatures. The physical adsorption of HMDS can also be disregarded since the samples were pre-washed with ethanol and deionized water. According to Equation (3), the grafting ratio of S_10HMDS_, S_15HMDS_, S_20HMDS_ and S_25HMDS_ can be calculated as 26%, 32%, 34%, and 39%, respectively.

In order to react with carboxyl acid-functionalized Al_2_O_3_ nanoparticles, further modification of HMDS-modified SiO_2_ was carried out using KH230 to graft −CH_2_Cl groups onto its surface. Figure 5c shows the XPS full survey spectra of o-S and S_25HMDS-KH230_ and the Si2p signal at 103.5 eV reveals the existence of pure SiO_2_. The O1s spectra signal peak absorbed at 532.3 eV clearly indicates the higher contribution of oxygen with silica. A small hump near 284 eV indicates the presence of residual carbon in the sample, potentially originating from carbon contamination within the instrument itself, similar to Al_2_O_3_. Apart from the above-mentioned elements, a Cl2p signal peak was detected at 199.8 eV in the sample S_25HMDS-KH230_, and there was a notable increase in the C1s signal due to the modification of the silica surface by the modifier. The single peak at 103.6 eV in the Si2p spectra of o-S was assigned to the Si-O bonds in SiO_2_ nanoparticles (Figure 5d). After modification, two peaks centered at 103.6 and 103 eV were identified in the Si2p spectra of S_25HMDS-KH230_, attributed to the Si-O and Si-C bonds, respectively. The deconvolution of C1s spectra of S_25HMDS-KH230_ shown in Figure 5e suggested the presence of C-C, C-Si and C-Cl bonds, with peaks at 285.1, 284.2 and 287 eV, respectively. These results suggest that KH230 is grafted onto the surface of HMDS-modified SiO_2_ nanoparticles to introduce −CH_2_Cl functional groups.

### 3.4. Coupling Reaction of Asymmetric Al_2_O_3_-SiO_2_ Janus Nanoparticles

**Figure 6:**
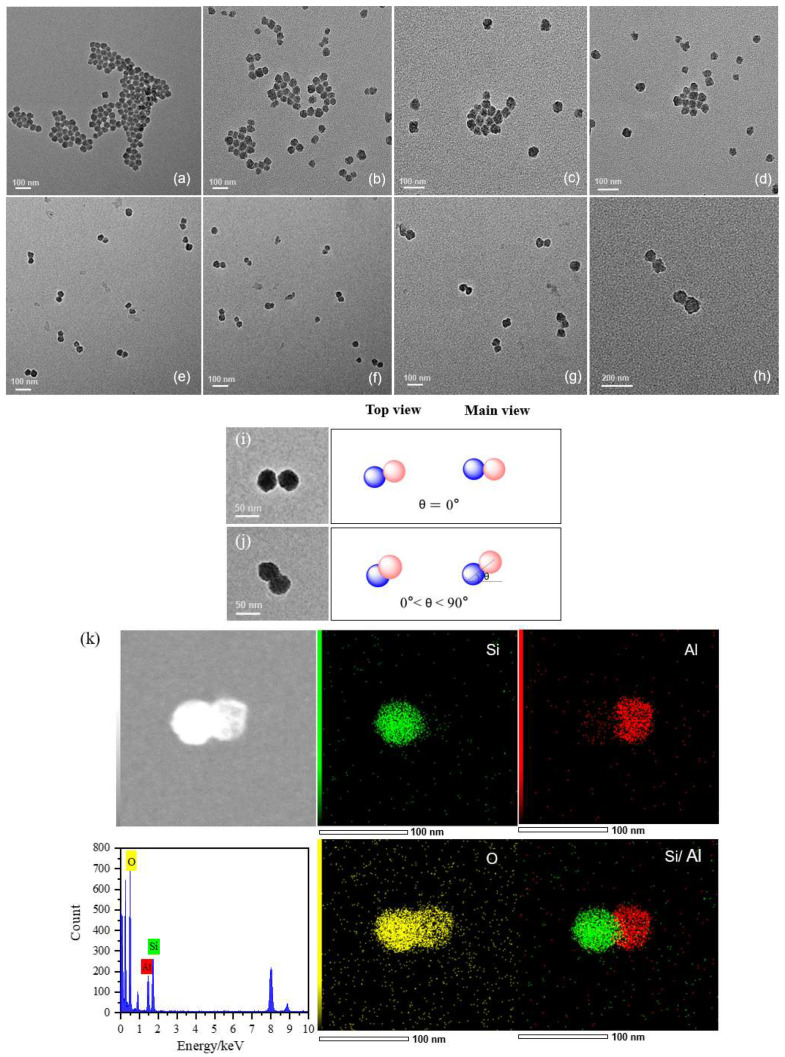
TEM images of (**a**) o-A; (**b**) o-S; (**c**) A_25KH550_-COOH @ o-S; (**d**) A_25KH550_-COOH @ S_25HMDS_ (with absence of KH230); (**e**–**h**) A_25KH550_-COOH @ S_25HMDS-KH230_ in different positions on the background; (**i**,**j**) different postures of A_25KH550_-COOH @ S_25HMDS-KH230_ in the TEM image (blue and light pink represent Al_2_O_3_ and SiO_2_ nanoparticles, respectively); (**k**) EDS mapping and EDS spectrum of dumbbell-like Al_2_O_3_-SiO_2_ nanoparticles (A_25KH550_-COOH @ S_25HMDS-KH230_).

The morphology of Al_2_O_3_ and SiO_2_ nanoparticles before and after coupling was investigated by TEM, as shown in Figure 6. The o-A and o-S are all uniformly spherical and well-shaped nanoparticles, but there was some obvious coalescence between them (Figure 6a,b). This can be attributed to the high specific surface area of Al_2_O_3_ and SiO_2_ nanoparticles and the large number of −OH groups distributed on their surfaces, making them prone to adhesion to each other. However, when the two types of nanoparticles were covalently connected in a one-to-one manner in the aqueous phase, the Al_2_O_3_-SiO_2_ nanoparticles with a dumbbell-like structure (A_25KH550_-COOH@S_25HMDS-KH230_) were clearly observed in different positions on the background (Figure 6e–h), which has different postures in the TEM images since they were randomly deposited on the background. Usually, the shooting angle of TEM is the top view. After the sample is dropped on the copper mesh and dried using an infrared lamp, the dispersant evaporates rapidly, causing the random deposition of dumbbell-like Al_2_O_3_-SiO_2_ nanoparticles onto the copper mesh background. Consequently, the nanoparticles display diverse orientations on the copper mesh surface, as depicted in Figure 6i,j. Moreover, the elements of Si and Al are independently distributed in the SiO_2_ and Al_2_O_3_ ends of the dumbbell-like Janus nanoparticles (as illustrated in Figure 6k), while O elements are dispersed throughout the entire structure of the dumbbell-like Al_2_O_3_-SiO_2_ nanoparticles, suggesting that these Janus nanoparticles are composed of spheroidal Al_2_O_3_ and SiO_2_ nanoparticles. In the comparative experiments, hydrophilic nanoparticles A_25KH550_-COOH were mixed with o-S (A_25KH550_-COOH @ o-S, Figure 6c). Severe agglomeration of the two nanoparticles was observed and no dumbbell-like structure was formed due to the absence of one of the reactive groups in the mixture. The same phenomenon was observed in another controlled experiment (Figure 6d), where hydrophilic nanoparticles A_25KH550_-COOH were mixed with hydrophobic nanoparticles S_25HMDS_ without prior modification with KH230. Furthermore, no multiple structures were observed in Figure 6e–h, which is closely related to the steric hindrance effect in the dumbbell structure.

From the above analysis on the morphology of Al_2_O_3_ and SiO_2_ nanoparticles before and after coupling, the two nanoparticles were covalently connected on a one-to-one basis through chemical bonds among surface groups to form dominant dumbbell-like nanoparticles and further reaction with other nanoparticles can be prevented by the strong steric hindrance around the dominant nanoparticles. The steric hindrance on the surface of nanoparticles is primarily imparted by the modifier, which condenses with −OH groups on the nanoparticle surface after modification, weakening the hydrogen bonding between particles and increasing the steric hindrance among them. Consequently, the dumbbell-like Al_2_O_3_-SiO_2_ Janus nanoparticles reached a thermodynamically stable state after the initial coupling reaction. Thus, the predominant entities within this reaction system are the dumbbell-like Al_2_O_3_-SiO_2_ Janus nanoparticles, aligning with our previous research findings [29,30]. Other studies have also demonstrated the critical role of steric hindrance in preventing the formation of multiple structures, such as the synthesis of dissymmetrical snowman and dumbbell-like silica/polymer colloidal particles through emulsion polymerization [16], and the assembly of DNA-linked colloidal gold NP building blocks for programmable matter [36], which further indicates the importance of steric hindrance in the formation of the asymmetric Al_2_O_3_-SiO_2_ Janus nanoparticles with a dumbbell-like structure.

**Figure 7:**
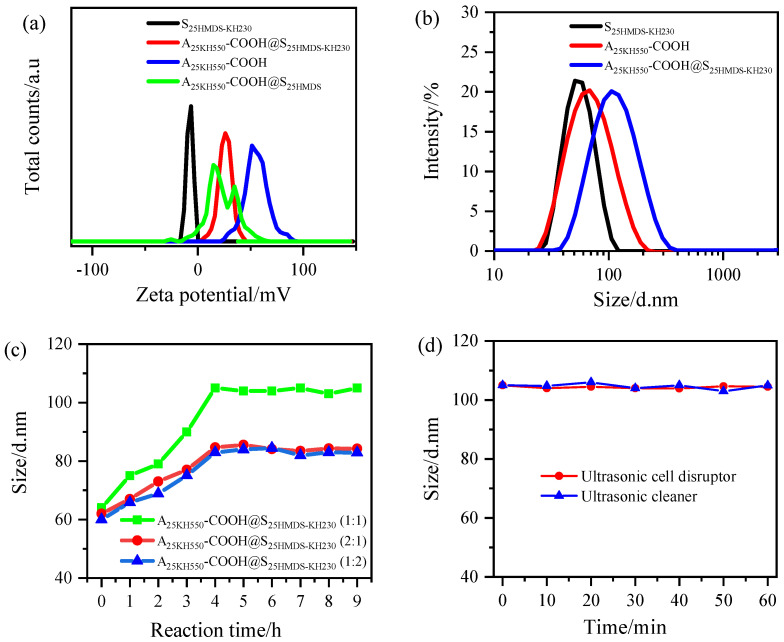
(**a**) Zeta potential of S_25HMDS-KH230_, A_25KH550_-COOH, A_25KH550_-COOH@ S_25HMDS-KH230_, and A_25KH550_-COOH@ S_25HMDS._ (**b**) Particle size distribution curves for Al_2_O_3_ and SiO_2_ nanoparticles before and after coupling. (**c**) Particle size evolution of A_25KH550_-COOH mixed with S_25HMDS-KH230_ in different ratios within 9 h. (**d**) The particle size evolution of A_25KH550_-COOH@S_25HMDS-KH230_ (1:1) under treatment of ultrasonic cell disruptor and ultrasonic cleaner within 60 min, respectively.

The formation of asymmetric Al_2_O_3_-SiO_2_ nanoparticles can be indirectly proved by the change in zeta potential (ZP) of Al_2_O_3_ and SiO_2_ nanoparticles before and after coupling. As shown in Figure S2, the ZP of o-A and o-S before modification was approximately 42 mV and −30 mV, respectively, indicating a stable system. After modification, the ZP of A_25KH550_ increased to about 54 mV, while the ZP of S_25HMDS_ changed from −30 mV to −9 mV. The results showed that −NH_2_ groups and −CH_3_ groups were successfully grafted on the surface of Al_2_O_3_ and SiO_2_ nanoparticles, respectively. The ZP changes after further functionalization of Al_2_O_3_ and SiO_2_ nanoparticles are shown in Figure 7a. The ZP values of S_25HMDS-KH230_ and A_25KH550_-COOH were −8 and 43 mV, respectively. A 1:1 mixture of the two nanoparticles produced dumbbell-like nanoparticles with a ZP value of 26 mV. Compared with the carboxylic acid-functionalized Al_2_O_3_ nanoparticles, the ZP value of the dumbbell-like Al_2_O_3-_SiO_2_ nanoparticles decreased significantly, which can be attributed to the reaction of the positively charged −NH_2_ groups on the surface of carboxylic-functionalized Al_2_O_3_ nanoparticles with the −CH_2_Cl groups on the hydrophobic SiO_2_ nanoparticles. The positively charged −NH_2_ groups are deprotonated due to the nucleophilic substitution with the −CH_2_Cl groups, so the ZP value of the A_25KH550_-COOH@S_25HMDS−KH230_ decreased. However, the zeta potential distribution of A_25KH550_-COOH@S_25HMDS_ exhibited a multi-peak distribution in the controlled experiment, indicating that the system was a mixed system without a coupling reaction. It can be concluded that the asymmetric Al_2_O_3_-SiO_2_ Janus nanoparticles with the dumbbell-like structure were successfully prepared via the nucleophilic substitution reaction of −NH_2_ and −CH_2_Cl groups.

Figure 7b illustrates the particle size distribution of samples obtained by mixing equal amounts of two nanoparticle hydrosols. The average particle size of A_25KH550_-COOH@S_25HMDS-KH230_ increased from 60 ± 1 nm to 105 ± 1 nm, while the PDI value remained below 0.17, indicating good monodispersity of the coupled nanoparticles in the aqueous phase, which is beneficial for study of particle size changes before and after coupling. The particle size evolution of A_25KH550_-COOH@S_25HMDS-KH230_ prepared under different mass ratios within 9 h was also investigated and is shown in Figure 7c. The particle size of all samples increased immediately with the increase in reaction time, and reached a stable value after 4 h, indicating that the coupling reaction was almost complete. Interestingly, even when one of the modified nanoparticles was present in excess, the measured particle sizes of the 2:1 and 1:2 ratios (about 84 nm) were smaller than the value of the 1:1 ratio (about 105 nm), suggesting that excess nanoparticles did not participate in the reaction but instead decreased the average particle size of the system along with the dumbbell-like Al_2_O_3_-SiO_2_ nanoparticles formed. These findings imply that the coupling reaction occurred between modified Al_2_O_3_ and SiO_2_ nanoparticles in a one-to-one manner through a chemical bond, and no higher nanoparticle structures were formed. To distinguish the chemically bonded Al_2_O_3_-SiO_2_ nanoparticles from physically attached ones, two kinds of sonication instruments, an ultrasonic cell disruptor (TL92-IID, Tianling Co., Ltd., Yancheng, China) and ultrasonic cleaner (SCIENTC, SB25-12DT, Ningbo, China), were applied to treat sample 6. As depicted in Figure 7d, the particle size of A_25KH550_-COOH@S_25HMDS-KH230_ (1:1) remained stable throughout the 60 min of sonication treatment. This indicates that there is a chemical bond between the Al_2_O_3_ and SiO_2_ nanoparticles and hard agglomeration cannot be disrupted by simple mechanical forces [37].

The results of the particle size distribution of different proportions of modified Al_2_O_3_ and SiO_2_ nanoparticles mixed in specific mass ratios, along with three control experiments, are summarized in Table 2. The average particle size of samples 1 to 6 increased gradually from 86 ± 1 nm to 105 ± 1 nm, which is related to the increase in reactive groups on the surface of the two nanoparticles. Compared to sample 6, the average particle sizes of samples 7 and 8 were about 86 and 84 nm, respectively, which are similar to those of samples 1 and 2, indicating that the system with few reactive groups behaves similarly to the system with an excess of one of the modified nanoparticles, resulting in an incomplete coupling reaction and a reduction in the overall average particle size. In the control sample 9 and sample 10, the average particle size measured was approximately 66 nm. In another controlled experiment (sample 11), A_25KH550_-COOH was mixed with S_25HMDS_ without KH230 modification under the same reaction conditions and the average particle size was measured to be 68 ± 1 nm. These results suggest that no coupling reaction occurred between A_25KH550_-COOH and o-S or o-A and S_25HMDS_ or A_25KH550_-COOH and S_25HMDS_ in the water system. It is worth noting that KH230 plays a critical bridging role in forming the asymmetric Al_2_O_3_-SiO_2_ Janus nanoparticles.

**Figure 8:**
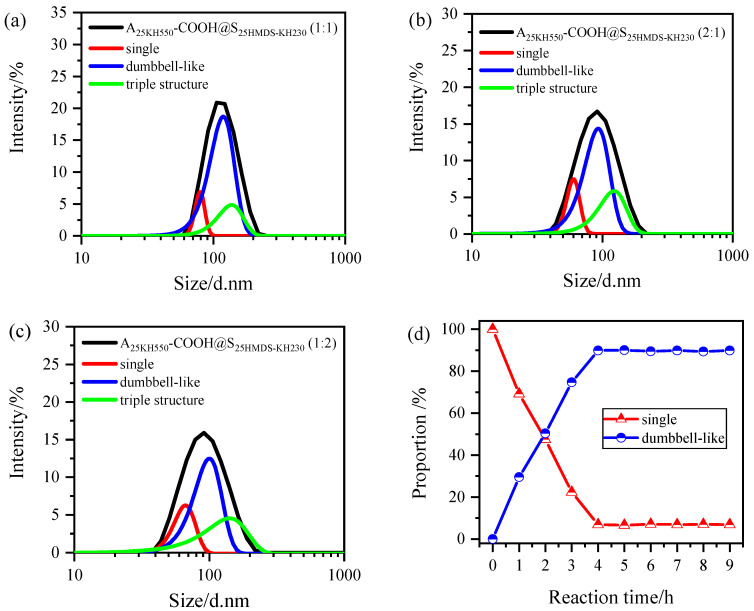
(**a**–**c**) Peak fitting analysis of the particle size distribution of dumbbell-like Al_2_O_3_-SiO_2_ nanoparticles (A_25KH550_-COOH@ S_25HMDS-KH230_): (a.1:1, b.2:1, c.1:2); (**d**) The proportion of single nanoparticles and dumbbell-like structures nanoparticles in A_25KH550_-COOH@S_25MDS-KH230_ with reaction time.

Based on our previous work [29], since the particle size distribution was analyzed by dynamic light scattering (DLS), the measurement particle size of anisotropic nanoparticles is expressed in terms of equivalent diameter. Therefore, the equivalent rod-like model proposed by Briard et al. [38] can be applied to the particle size measurement of asymmetric Al_2_O_3_-SiO_2_ Janus nanoparticles. The equivalent diameter can be calculated using Equation (4).

(4) $$d_{E}=2L\left[\frac{f}{f_{0}}\right]\;{\left(\frac{3}{{16p}^{2}}\right)}^{\frac{1}{3}}$$

where *L* and *p* represent the length and aspect ratio of the rod-like nanoparticles, respectively, $d_{E}$ is the equivalent diameter of the rod-like nanoparticles, *f* is the translational friction coefficient of the rod-like nanoparticle and *f_0_* is the translational friction coefficient of a sphere that has the same volume as the rod-like nanoparticle. In this study, it is assumed that the nanoparticle mixture after the coupling reaction contains three different components: unreacted single nanoparticles, coupled dumbbell-like nanoparticles, and triple structure nanoparticles, which are denoted as d_1_, d_2_, and d_3_, respectively. The previous measurement results obtained by DLS have shown that the particle sizes of Al_2_O_3_ (d_A1_) and SiO_2_ (ds_1_) are approximately equal to 60 nm, so it can be succinctly considered that *d_A_*_1_ *= ds*_1_ *=d*. Finally, *d*_1_ *= d*, *d*_2_ *=* 1.6*d*, and *d*_3_ *=* 2*d* were obtained according to the previous study. Then, the peak fittings of the particle size distribution curves of the coupled Al_2_O_3_-SiO_2_ Janus nanoparticles were performed using the method from [29] and the coupling reaction between the two types of nanoparticles was quantitatively analyzed by combining Equations (5) and (6) inspired by Rayleigh Approximation Theory [39].

(5) $$\%I_{1}=\frac{N_{1}{d_{1}}^{6}\cdot 100}{N_{1}{d_{1}}^{6}+N_{2}{d_{2}}^{6}+N_{3}{d_{3}}^{6}}$$

(6) $$\%N_{1}=\frac{N_{1}\cdot 100}{N_{1}+N_{2}+N_{3}}$$

where size *d*_1_, *d*_2_, and *d*_3_ represent the diameter of single nanoparticles, dumbbell-like nanoparticles, and triple structure nanoparticles. *N*_1_, *N*_2_, and *N*_3_ are particle numbers with sizes d_1_, d_2_, and d_3_. In addition, %I_1_ and %N_1_ present intensity-weighted distribution and number-weighted distribution for small particles with size *d*_1_, based on the relative amount of intensity of particles and the number of particles with size d_1_, respectively. When A_25KH550_-COOH was mixed with S_25HMDS-KH230_ in a 1:1 ratio, the obtained intensity ratios of single nanoparticles, coupled dumbbell-like nanoparticles, and triple structure nanoparticles were plotted in Figure 8a, which were 0.7% (%I_1_), 89.2% (%I_2_), and 10.1% (%I_3_), respectively. Next, by combing Equations (5) and (6) with the values of the intensity ratios of the different nanoparticles, the ratios *N*_1_:*N*_2_ = 0.15:1, and *N*_3_:*N*_2_ = 0.03:1 were obtained. The asymmetric Al_2_O_3_-SiO_2_ Janus nanoparticles with a dumbbell-like structure accounted for about 90% of the total number of nanoparticles in system after the coupling reaction, whereas the triple structure accounted for only 3.2% (results are shown in Table 3). When the two nanoparticles were mixed in a 2:1 or 1:2 ratio, the particle size distribution of the samples was also analyzed by the same method, as shown in Figure 8b,c and the proportions of coupled Al_2_O_3_-SiO_2_ Janus nanoparticles with a dumbbell-like structure were 61.4% and 67.5%, respectively, while that of triple structure nanoparticles were 5.9% and 5.6%, respectively. This indicated that a relatively complete transformation from single nanoparticles to dumbbell-like nanoparticles occurred in a 1:1 hybrid water system compared with 2:1 or 1:2, in which the higher structures, such as triple structure nanoparticles, are negligibly small.

In addition, the effect of the amounts of modifiers on the coupling reaction in a 1:1 hybrid water system was also investigated in Figure S3a–e, and the obtained results are listed in Table 3. The proportion of Al_2_O_3_-SiO_2_ nanoparticles with a dumbbell-like structure in the total number of nanoparticles in the system after the coupling reaction increased with the increase in modifier dosage, ranging from 56% to 82%, indicating that the amount of modifier is related to the reactive groups on the surface of the nanoparticles. Specifically, a lower amount of modifier resulted in fewer single nanoparticles participating in the coupling reaction, while a higher amount of modifier led to a higher transformation rate from single nanoparticles to dumbbell-like nanoparticles. It can be concluded that the dominant structure in the coupling reaction is the dumbbell-like structure, while the proportion of triple structure nanoparticles is very small. Therefore, the presence of higher structures, such as quadruple structure nanoparticles, can be disregarded.

Furthermore, the peak fitting process was conducted on the particle size distribution curves of different reaction times using sample 6 (A_25KH550_-COOH@S_25MDS-KH230_) as an example to investigate the changes in the proportion of single nanoparticles and dumbbell-like structures as the reaction time increased. The results are depicted in Figure 8d. It was observed that the proportion of single nanoparticles decreased significantly within 4 h, accompanied by a noticeable increase in the proportion of dumbbell-like structures. After 4 h, the reaction was essentially completed, with approximately 90% of the single nanoparticles transformed into dumbbell-like nanoparticles.

### 3.5. Application of Asymmetric Al_2_O_3_-SiO_2_ Janus Nanoparticles in Surfactant

**Figure 9:**
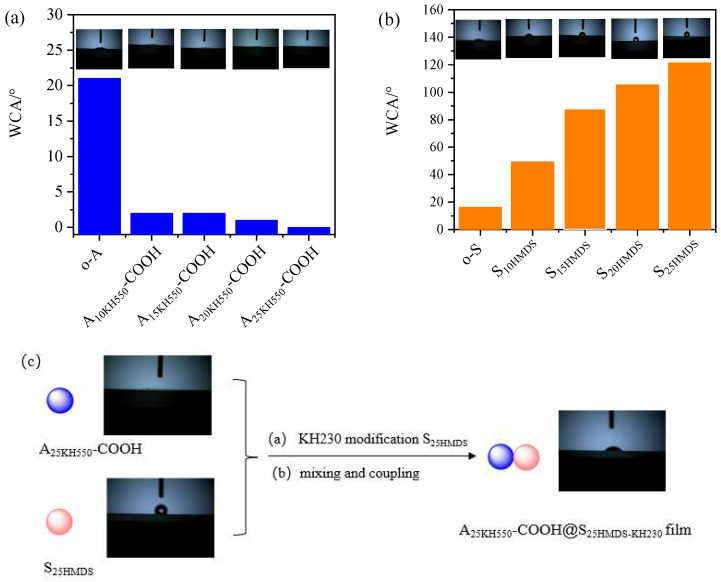
Water contact angles (WCAs) of (**a**) carboxylic-functionalized Al_2_O_3_ nanoparticles, (**b**) HMDS-modified SiO_2_ nanoparticles, and (**c**) asymmetric Al_2_O_3_-SiO_2_ Janus nanoparticles (A_25KH550_-COOH@S_25HMDS-KH230_).

The hydrophilicity of nanoparticles was characterized by water contact angle (WCA) measurement, as presented in Figure 9. Before measuring the WCA, Al_2_O_3_ and SiO_2_ nano hydrosol must be deposited onto the silicon wafer using a simple sol–gel dip-coating process. Detailed experimental procedures are provided in Text S2. The WCA values of o-A, A_10KH550_-COOH, A_15KH550_-COOH, A_20KH550_-COOH, and A_25KH550_-COOH were 21°, 2°, 2°, 1°, and 0° (Figure 9a), indicating a superhydrophilic surface, while that of o-S, S_10HMDS_, S_15HMDS_, S_20HMDS_, and S_25HMDS_ were 16°, 49°, 88°, 105°, and 121°, respectively (Figure 9b). The WCA values exhibited significant changes with an increasing HMDS content. When the HMDS content reached 25% of the mass of SiO_2_ in hydrosol, the WCA reached 121°, indicating a better hydrophobic modification of SiO_2_ under these conditions. Therefore, two types of nanoparticles with significant differences in hydrophilicity were obtained after modification. The superhydrophilic A_25KH550_-COOH and hydrophobic S_25HMDS_ were then coupled to obtain asymmetric Al_2_O_3_-SiO_2_ Janus nanoparticles (A_25KH550_-COOH@S_25HMDS-KH230_) with a WCA of 44° (Figure 9c). Evidently, A_25KH550_-COOH@S_25HMDS-KH230_ possess an amphiphilic property in addition to chemical asymmetry. As a result, these nanoparticles can be regarded as amphiphilic Janus nanoparticles to emulsify the oil–water system.

**Figure 10:**
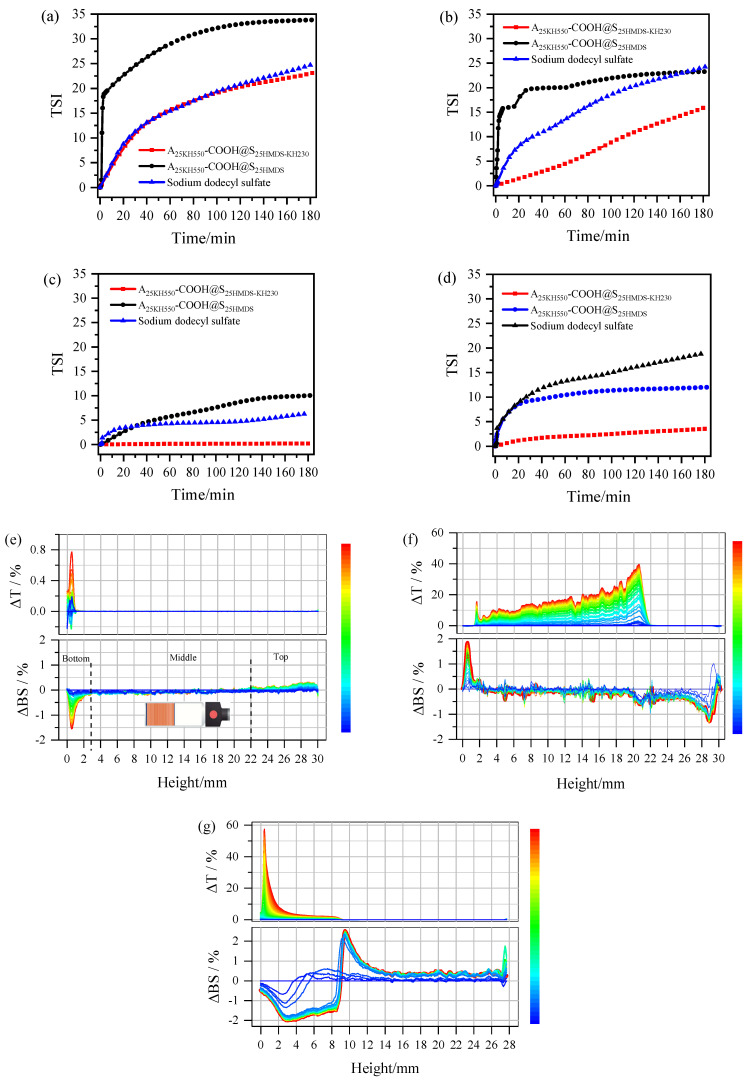
(**a**–**d**) Change in TSI value with time in different oil–water systems after emulsification by A_25KH550_-COOH@S_25HMDS-KH230_, A_25KH550_-COOH@S_25HMDS_ and sodium dodecyl sulfate: (**a**) toluene–water, (**b**) cyclohexane–water, (**c**) silicone oil–water, (**d**) vegetable oil–water); (**e**–**g**) delta transmission (ΔT, top) and delta backscattering profiles (ΔBS, bottom) of silicone oil–water systems after emulsification by A_25KH550_-COOH@S_25HMDS-KH230_ (**e**), A_25KH550_-COOH@S_25HMDS_ (**f**) and sodium dodecyl sulfate (**g**) (time axis: blue to red represents from 0 to 3 h).

The amphiphilicity of asymmetric Al_2_O_3_-SiO_2_ Janus nanoparticles was tested using A_25KH550_-COOH@S_25HMDS-KH230_ to stabilize the oil–water system. Different model compounds such as cyclohexane–water, toluene–water, silicone oil–water, and vegetable oil–water were used for this purpose. Digital photographs of samples suspended in a dual-phase mixture of various oils and water are presented in Figure S4 and o-A, A_25KH550_-COOH, and o-S dispersed only in water as a result of their hydrophilic hydroxyl groups or carboxyl groups, while S_25HMDS_ was mainly dispersed in oil due to the introduction of hydrophobic alkyl groups. In a control experiment, the oil–water emulsions were not stabilized by the simple physical mixture (A_25KH550_-COOH@S_25HMDS_), which was separated into two layers within 5 min. However, A_25KH550_-COOH@S_25HMDS-KH230_ could effectively stabilize emulsions of different oil and water mixtures, forming a stable emulsion layer with a thickness of 3.2cm. The effect of amphiphilic asymmetric Al_2_O_3_-SiO_2_ Janus nanoparticles on the stability of four oil–water emulsions was then further evaluated by Turbiscan based on the backscattering technology [40,41]. Given that one end of Al_2_O_3_-SiO_2_ Janus nanoparticles exhibits anion properties, a common anionic surfactant, namely sodium dodecyl sulfate, was selected for comparison with A_25KH550_-COOH@S_25HMDS-KH230_ and A_25KH550_-COOH@S_25HMDS_. The Turbiscan Stability Index (TSI) curves for different emulsions are depicted in Figure 10. The changes in the TSI values of emulsions indicated physical stability during storage. The higher the TSI value, the lower the stability of the system [42]. The TSI curves of various oil–water systems emulsified by A_25KH550_-COOH@S_25HMDS-KH230_ consistently exhibit lower values compared to those of sodium dodecyl sulfate and A_25KH550_-COOH@S_25HMDS_, except for the similarity in emulsification capabilities between A_25KH550_-COOH@S_25HMDS-KH230_ and sodium dodecyl sulfate within the initial 2 h, as observed in Figure 10a. Table 4 presents the TSI values of different oil–water systems at 3 h and the stability provided by A_25KH550_-COOH@S_25HMDS_ and sodium dodecyl sulfate for the four oil–water systems is inferior to that of A_25KH550_-COOH@S_25HMDS-KH230_. These results indicated that the dumbbell-like Al_2_O_3_-SiO_2_ Janus nanoparticles with amphiphilic properties have a good affinity for both water and oil and exhibit significantly higher interfacial activity than their homogeneous nanoparticles, effectively lowering the interfacial tension between oil and water.

To further characterize the physical stability of emulsions, Turbiscan delta transmission (ΔT) and delta backscattering (ΔBS) data were plotted against sample height over time (Figure 10e–g, using silicone oil–water emulsions as an example). For the silicone oil–water systems emulsified by A_25KH550_-COOH@S_25HMDS-KH230_ (Figure 10e), the change in ΔT signal increased between 0 and 1 mm over the entire height of the sample during the scan (3 h), indicating that this zone was starting to become clear. However, the T signal remained close to zero in the middle and top of the samples, indicating a high degree of emulsion stability with no apparent demulsification. Conversely, between 0 and 3 mm heights at the bottom of the sample vials, an apparent decrease in the BS signal was observed, suggesting slight flocculation or coalescence-induced sedimentation in silicone oil–water emulsions during storage [43]. No obvious changes in backscattered light were observed in the middle of the sample, indicating no droplet size change phenomenon. The BS signal at the top of sample vials increased with time due to the formation of the upper emulsion layer. In the case of A_25KH550_-COOH@S_25HMDS_ (Figure 10f), an obvious increase in the T signal was observed in the 0–22 mm region from the bottom to the middle with the formation of a thicker water-clearing layer in that area, indicating the significant stratification and instability of the system. The increase in the BS signal in the bottom indicated a higher droplet concentration in the emulsion layer, whereas the decrease in the BS signal at the top regions indicated a lower droplet concentration. Similarly, in the silicone oil–water systems emulsified by sodium dodecyl sulfate (Figure 10g), a layer thickness of around 9 mm was observed at the top in the ΔT spectrum. Correspondingly, the BS signal decreased in the bottom regions (0–9 mm), while it increased in the middle region (9–14 mm) and top region (14–28 mm), indicating the formation of a water layer at the bottom. Furthermore, the T and BS changes in Figure 10e,f are basically identical, so the emulsions obtained are of the same type, that is, O/W emulsion. The ΔT and ΔBS profiles of the other oil–water systems emulsified by A_25KH550_-COOH@S_25HMDS-KH230_, A_25KH550_-COOH@S_25HMDS_ and sodium dodecyl sulfate are shown in Figures S5–S7 and the analytical methods of T and BS spectra are the same as above. Collectively, the clarification at the bottom of the sample is associated with the increase in the T signal, while the droplet concentration (emulsification) at the top of the sample was linked with an increased BS signal. These changes in TSI, T data, and BS data of the emulsions suggested that the asymmetric Al_2_O_3_-SiO_2_ Janus nanoparticles with dumbbell-like structures exhibit significantly enhanced interfacial activity. This enhancement effectively reduces the interfacial tension between oil and water, highlighting their potential as promising surfactants for emulsifying certain oil–water emulsions.

## 4. Conclusions

In this study, asymmetric Al_2_O_3_-SiO_2_ Janus nanoparticles with dumbbell-like structures were successfully fabricated in an aqueous phase by coupling carboxyl-functionalized Al_2_O_3_ nanoparticles and hydrophobic SiO_2_ nanoparticles through substitution reactions of −NH_2_ and −CH_2_Cl groups on the surface of the nanoparticles. TEM analysis confirmed the presence of a dumbbell structure in Al_2_O_3_-SiO_2_ Janus nanoparticles, while FTIR, XPS and WCA measurements confirmed the distinct surfaces and functionalities of these nanoparticles. Detailed DLS analysis indicated that the transformation from single nanoparticles to dumbbell-like nanoparticles predominantly took place within the 1:1 hybrid system and more than 85% of the single nanoparticles (Al_2_O_3_ and SiO_2_) transformed to novel dumbbell-like structures. As the amount of modifier increased, the proportion of dumbbell-like structures among the total number of nanoparticles after the coupling reaction increased from 56% to 82%. Emulsification tests showed that the dumbbell-like Al_2_O_3_-SiO_2_ Janus nanoparticles exhibit significantly higher interfacial activity than their homogeneous nanoparticles. These findings highlight the promising potential of dumbbell-like Al_2_O_3_-SiO_2_ Janus nanoparticles in surfactant applications, owing to their unique amphiphilic interfacial properties.

Additionally, the Al_2_O_3_ lobes in Janus nanoparticles exhibit unique anionic properties compared to those prepared in our previous studies. This feature facilitates directional self-assembly on functionalized substrates, potentially offering valuable insights for further research on the application of dumbbell-shaped Al_2_O_3_-SiO_2_ Janus nanoparticles in film layers. Overall, this fabrication method not only enables large-scale production of various asymmetric Janus nanoparticles using pure inorganic materials but also provides relatively precise control over the structure and composition of Janus nanoparticles, which is of great significance for systematically studying the performance of Janus particles and further exploring its practical applications, such as emulsification, environmental remediation, catalysis and functional coatings and films with tailored properties. However, further research is needed to optimize the properties of these Janus nanoparticles and develop cost-effective manufacturing processes.

## Figures and Tables

**Table 1:** Titration results of carboxylic acid-functionalized Al_2_O_3_ nanoparticles and SA conversion rate.

Sample	m_sample_ (g)	C_NaOH_ (mol L^−1^)	V_NaOH_ (mL)	${\mathrm{Grafting}\;\mathrm{Ratio}\;}_{\mathrm{SA}}^{\;a}$ (%)	Conversion Rate of SA (%)
A_0KH550_-COOH	0.2	0.004	0.3	0.3	0.9
A_10KH550_-COOH	0.2	0.004	17.3	17	76.9
A_15KH550_-COOH	0.2	0.004	26.8	27	78.8
A_20KH550_-COOH	0.2	0.004	36.1	36	80.2
A_25KH550_-COOH	0.2	0.004	44.2	44	80.4

Note: The result in the table is the average of the three titrations.

**Table 2:** Particle size and PDI values of dumbbell-like Al_2_O_3_-SiO_2_ nanoparticles (1−8) and samples in three control experiments (9–11).

Sample	Types of Nanoparticles		Mixing Ratio	After Mixing for 4 h	
	Hydrophilic	Hydrophobic		Size/d. nm	PDI
1	A_3KH550_-COOH	S_3HMDS-KH230_	1:1	86 ± 1	<0.18
2	A_5KH550_-COOH	S_5HMDS-KH230_	1:1	89 ± 1	<0.16
3	A_10KH550_-COOH	S_10HMDS-KH230_	1:1	92 ± 1	<0.18
4	A_15KH550_-COOH	S_15HMDS-KH230_	1:1	96 ± 1	<0.19
5	A_20KH550_-COOH	S_20HMDS-KH230_	1:1	97 ± 1	<0.18
6	A_25KH550_-COOH	S_25HMDS-KH230_	1:1	105 ± 1	<0.17
7	A_25KH550_-COOH	S_25HMDS-KH230_	2:1	86 ± 1	<0.23
8	A_25KH550_-COOH	S_25HMDS-KH230_	1:2	84 ± 1	<0.22
9	A_25KH550_-COOH	o-S	1:1	66 ± 1	<0.17
10	o-A	S_25HMDS_	1:1	66 ± 1	<0.18
11	A_25KH550_-COOH	S_25HMDS_	1:1	68 ± 1	<0.18

**Table 3:** The proportions of nanoparticles with different structures after coupling reaction.

Sample	Types of Nanoparticles		Mass Mixing Ratio	Proportion of Nanoparticles with Different Structures after Coupling Reaction (%)		
	Hydrophilic	Hydrophobic		Single	Dumbbell-like	Triple
1	A_3KH550_-COOH	S_3HMDS-KH230_	1:1	39.3	56.6	4.1
2	A_5KH550_-COOH	S_5HMDS-KH230_	1:1	35.6	60.4	4.0
3	A_10KH550_-COOH	S_10HMDS-KH230_	1:1	23.2	73.1	3.7
4	A_15KH550_-COOH	S_15HMDS-KH230_	1:1	19.5	76.9	3.6
5	A_20KH550_-COOH	S_20HMDS-KH230_	1:1	14.8	81.8	3.4
6	A_25KH550_-COOH	S_25HMDS-KH230_	1:1	6.9	89.9	3.2
7	A_25KH550_-COOH	S_25HMDS-KH230_	2:1	32.7	61.4	5.9
8	A_25KH550_-COOH	S_25HMDS-KH230_	1:2	26.9	67.5	5.6

Note: single nanoparticles (%) = *N*_1_*/(N*_1_ *+* 2*N*_2_ *+* 3*N*_3_*)*, dumbbell-like nanoparticles (%) = 2*N*_2_*/(N*_1_ *+* 2*N*_2_ *+* 3*N*_3_*)* and triple structure nanoparticles (%) = 3*N*_3_*/(N*_1_ *+* 2*N*_2_ *+* 3*N*_3_*)*.

**Table 4:** TSI values of different oil–water systems at 3 h.

Sample	TSI			
	Toluene–Water	Cyclohexane–Water	Silicone Oil–Water	Vegetable Oil–Water
A_25KH550_-COOH@S_25HMDS-KH230_	24	15	1	4
Sodium dodecyl sulfate	25	25	6	12
A_25KH550_-COOH@S_25HMDS_	34	23	10	19

## Data Availability

The data presented in this study are available upon request (due to privacy).

## Supplementary Materials

The following supporting information can be downloaded at: https://www.mdpi.com/article/10.3390/ma17061251/s1, Figure S1: TGA curves of KH550 modified Al_2_O_3_ nanoparticles; Figure S2: Zeta potential of o-A, A_25KH550_, o-S and S_25HMDS_; Figure S3: Peak fitting analysis of the particle size distribution of various dumbbell-like Al_2_O_3_-SiO_2_ nanoparticles; Figure S4: Photographs of various nanoparticles stabilization of four oil-water model systems after stirring for 10min at room temperature; Figures S5–S7: Delta transmission (ΔT, top) and delta backscattering profiles (ΔBS, bottom) of oil-water systems after emulsification by A_25KH550_-COOH@S_25HMDS-KH230_, A_25KH550_-COOH@S_25HMDS_ and sodium dodecyl sulfate, respectively; Text S1: Titration of carboxyl groups on the surface of Al_2_O_3_ nanoparticles; Text S2: Preparation of Al_2_O_3_ and SiO_2_ film by sol-gel dip-coating process; Equation (S1)**:** Equation for determining the conversion rate (CR) of SA; Equation (S2): Equation for determining $n_{SA}$ in 1g of sample.
