# Supplementary material for: Preparation of Asymmetric Al2O3-SiO2 Janus Nanoparticles in Aqueous Phase and Its Interfacial Property
Source: Materials (Basel). 2024 Mar 8;17(6):1251. doi: 10.3390/ma17061251 (PMC10972201; doi:10.3390/ma17061251)
Supplement: Supplementary file 1 [file materials-17-01251-s001.zip › materials-2845166-supplementary.pdf]

Supporting Information

Preparation of asymmetric Al_2O_3-SiO_2 Janus nanoparticles in aqueous phase and its interfacial property

Xinli Jia [1], Peiwen Xiao [2,3], Liqiang Yang[4], Jianhui Luo [2,3], Meiying He[5], Pingmei Wang [2,3], Bo Jiang [1] and Bo Xiao [1], *

[1] Key Laboratory of Green Chemistry & Technology, Ministry of Education, College of Chemistry, Sichuan University, Chengdu 610064, China

[2] Research Institute of Petroleum Exploration & Development (RIPED), PetroChina, Beijing 100083, China

[3] Key Laboratory of Nano Chemistry (KLNC), China National Petroleum Corporation (CNPC), Beijing 100083, China

[4] PetroChina, Beijing 100007, China

[5] China Chengda Engineering Co., Ltd., Chengdu 610041, China

* Corresponding Author: xiaobo_chem@scu.edu.cn

Table of contents da rivedere

Text S1. 2
Equation S1.. 2
Equation S2.. 2
Text S2. 2
Figure S1. 3
Figure S2.. 3
Figure S3. 4
Figure S4. 5
Figure S5. 5
Figure S6. 6
Figure S7. 6

Text S1. Titration of carboxyl groups on the surface of Al_2O_3 nanoparticles.

Since carboxyl groups are acidic in water, sodium hydroxide solution was used to directly titrate the carboxyl groups on the surface of Al_2O_3 nanoparticles. Approximately 0.2 g of dried carboxylated xerogels was weighed using an analytical balance and placed into a 200 mL beaker, along with 50 mL of deionized water. The dried carboxylated xerogels are sufficiently dispersed in water by ultrasonic dispersion. The three drops of phenolphthalein solution were added to the mixture. The prepared solution was then titrated with a 0.004 M sodium hydroxide solution. The endpoint of the titration was reached when the solution turned red and remained unchanged for 1 minute.

Equation S1. Equation for determining the conversion rate (CR) of SA is shown as follows:

$$\mathrm{CR} = \frac{n_{SA} \times M_{SA}}{m_{total\ SA}} \times 100\% \qquad (S1)$$

Equation S2. Equation for determining n_{SA} in 1g of sample is shown as follows:

$$n_{SA} = \frac{C_{OH} \times V_{OH}}{m_{sample}} \times 1000 \times 1g \qquad (S2)$$

where n_{SA} is the molar mass of SA, C_{OH} is the concentration of NaOH standard solution, V_{OH} is the volume of NaOH standard solution needed to reach the equivalence point for the sample, m_{sample} is the mass of the sample, and $m_{total\ SA}$ is the total mass of SA. M_{SA} is the molecular weight of SA.

Text S2. Preparation of Al_2O_3 and SiO_2 film by sol-gel dip-coating process

Since the Al_2O_3 and SiO_2 hydrosols used in the experiment are highly polar water-based solutions, it is not suitable for coating onto the silicon wafer. Therefore, methanol with the second polarity was chosen to dilute the Al_2O_3 and SiO_2 hydrosols before and after modification, as well as the Al_2O_3+SiO_2 hydrosol after coupling. The freshly prepared alcohol hydrosols were

then deposited on the silicon wafer to form coatings via dip-coating process with a dip coater (SYDC-100, Shanghai Sanyan Technology, China). The ambient temperature and relative humidity (RH) were 30±2℃ and 35±5%. The resulting films were subsequently dried at 100℃ for 1h to allow complete solvent evaporation and obtain dry gels. The substrates were washed successively in dilute hydrofluoric acid (0.1 wt.%), deionized water and ethanol and wiped carefully before dip-coating process. The substrates were immersed in the alcohol hydrosols for five seconds and then lifted vertically at a constant withdraw rate. Finally, each coating was used to test the water contact angle (WCA) of the samples.

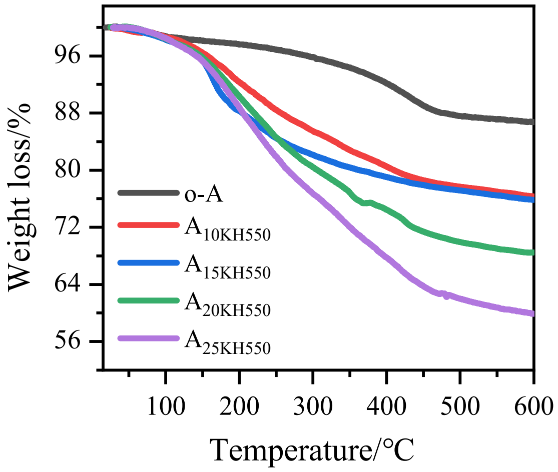

Figure S1. TGA curves of KH550 modified Al_2O_3 nanoparticles

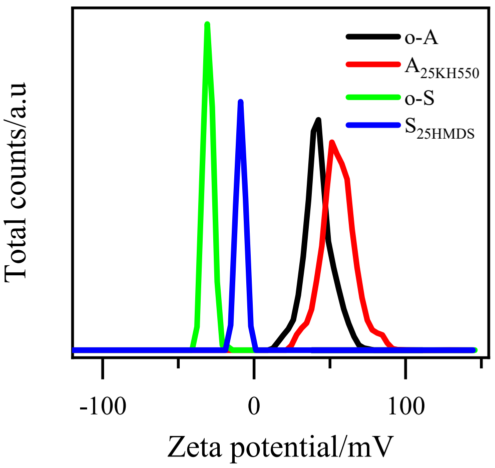

Figure S2. Zeta potential of o-A, $A_{25KH550}$, o-S and S_{25HMDS}.

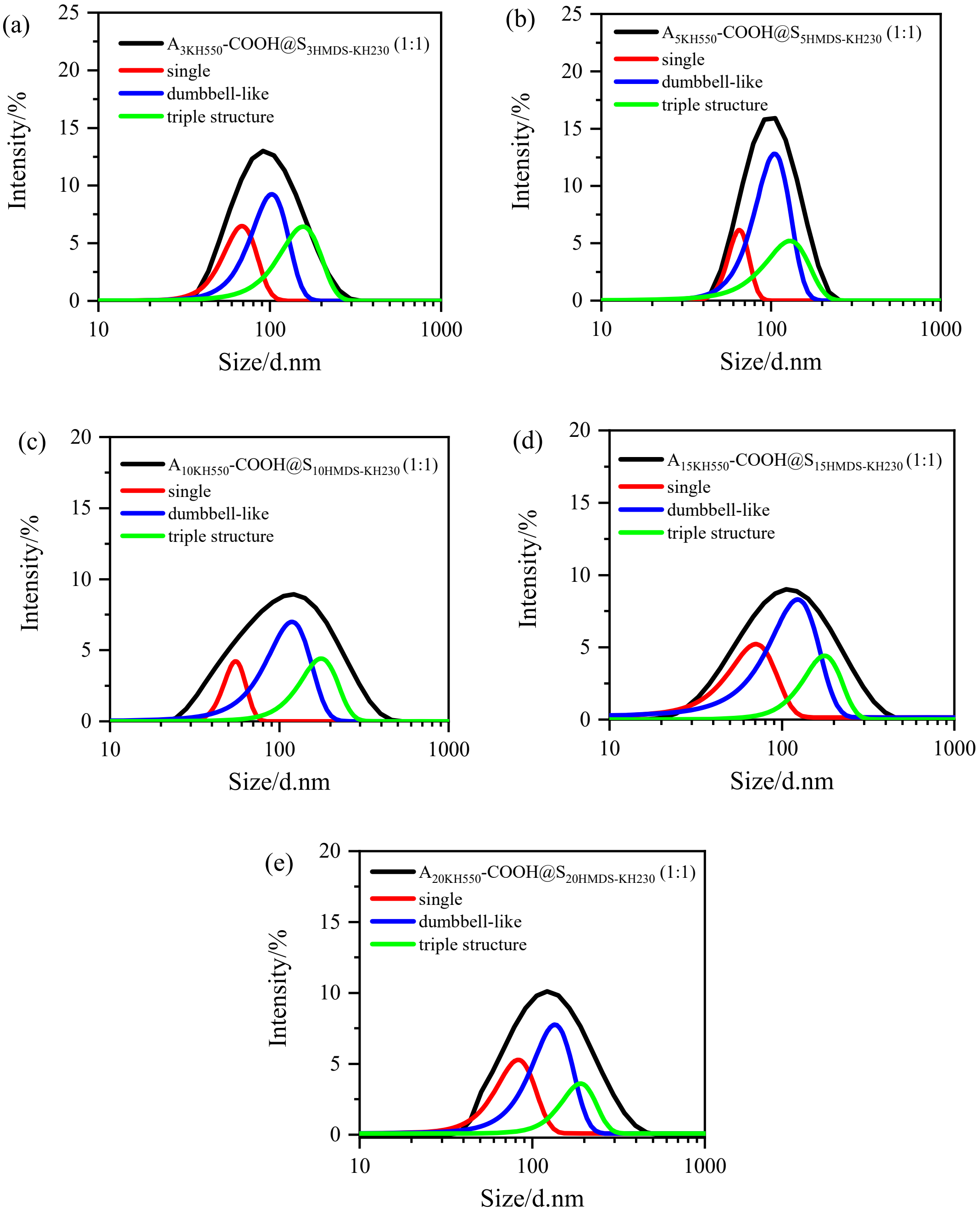

Figure S3. Peak fitting analysis of the particle size distribution of various dumbbell-like Al_2O_3-SiO_2 nanoparticles: (a) A_{3KH550}-COOH@$S_{3HMDS-KH230}$; (b) A_{5KH550}-COOH@$S_{5HMDS-KH230}$; (c) $A_{10KH550}$-COOH@$S_{10HMDS-KH230}$; (d) $A_{15KH550}$-COOH@$S_{15HMDS-KH230}$; (e) $A_{20KH550}$-COOH@$S_{20HMDS-KH230}$

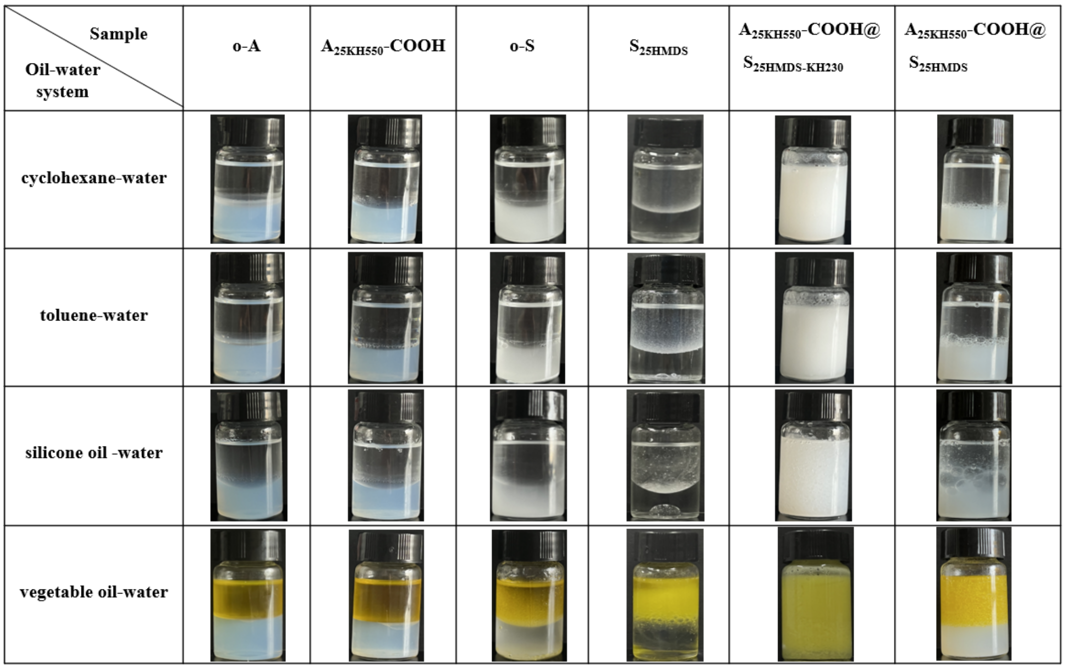

Figure S4. Photographs of various nanoparticles stabilization of four oil-water model systems after stirring for 10min at room temperature and standing for a moment (oil is the phase on top, water is the phase at the bottom, emulsifier dosage is 0.05g)

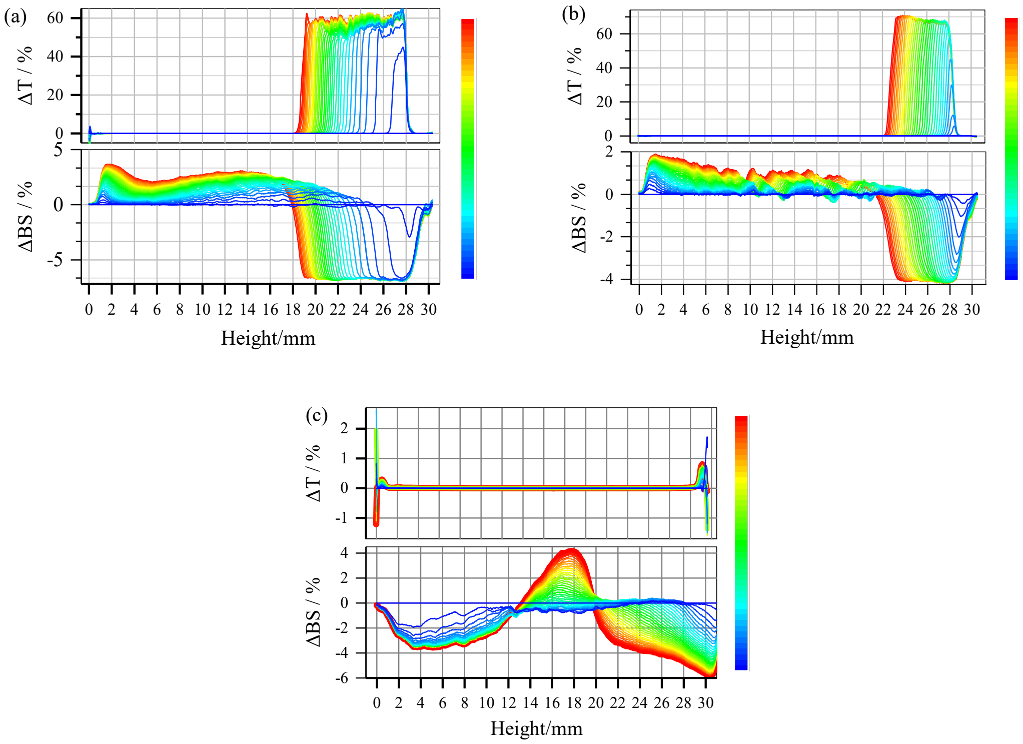

Figure S5. Delta transmission (ΔT, top) and delta backscattering profiles (ΔBS, bottom) of oil-water systems after emulsification by $A_{25KH550}$-COOH@$S_{25HMDS-KH230}$: (a) toluene-water emulsions; (b) cyclohexane-water emulsions; (c) vegetable oil-water emulsions (Time axis: blue to red represents from 0 to 3h)

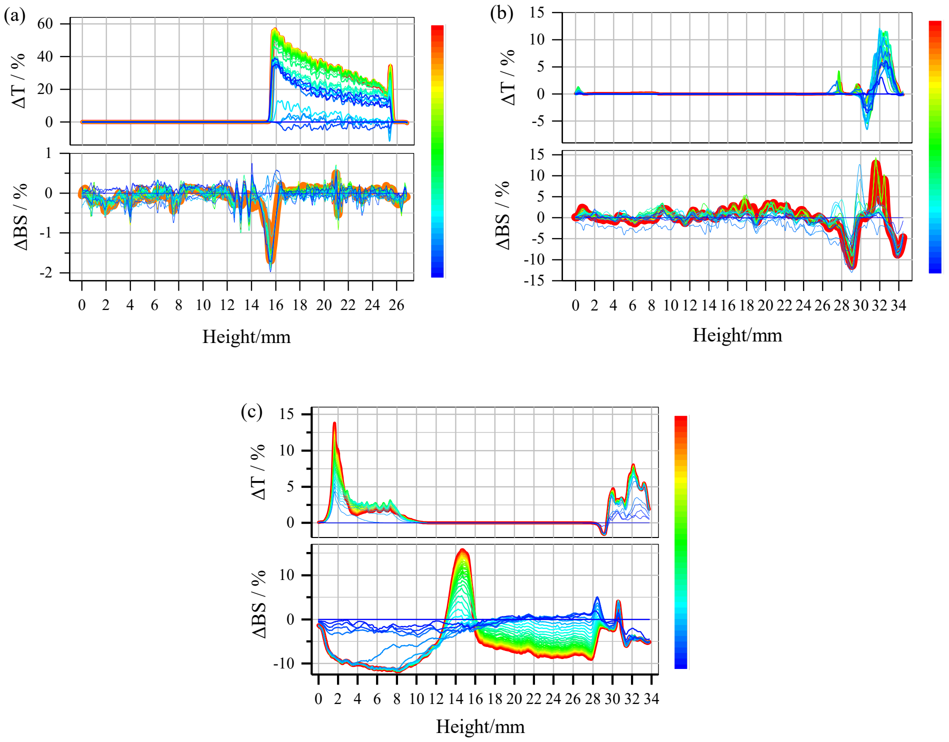

Figure S6. Delta transmission (ΔT, top) and delta backscattering profiles (ΔBS, bottom) of oil-water systems after emulsification by $A_{25KH550}$-COOH@S_{25HMDS}: (a) toluene-water emulsions; (b) cyclohexane-water emulsions; (c) vegetable oil-water emulsions (Time axis: blue to red represents from 0 to 3h)

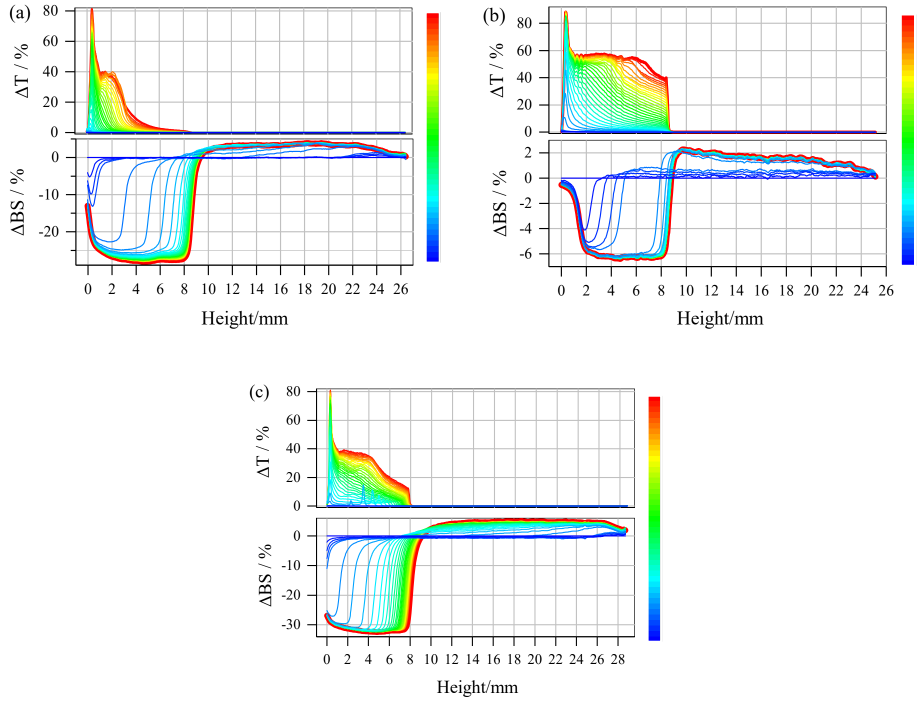

Figure S7. Delta transmission (ΔT, top) and delta backscattering profiles (ΔBS, bottom) of oil-

water systems after emulsification by sodium dodecyl sulfate: (a) toluene-water emulsions; (b) cyclohexane-water emulsions; (c) vegetable oil-water emulsions (Time axis: blue to red represents from 0 to 3h)
